# Development of an Immersive Virtual Reality-Based Nursing Program Involving Patients with Respiratory Infections

**DOI:** 10.3390/bioengineering13010098

**Published:** 2026-01-15

**Authors:** Eun-Joo Ji, Sang Sik Lee, Eun-Kyung Lee

**Affiliations:** 1 Department of Nursing, College of Medicine, Catholic Kwandong University, Gangneung, 24 Beomil-ro 579 Beongil, Gangneung-si 25601, Republic of Korea; 93eunjoo@cku.ac.kr; 2 Department of Digital Healthcare, Catholic Kwandong University, 24 Beomil-ro 579 Beongil, Gangneung-si 25601, Republic of Korea; lsskyj@cku.ac.kr; 3 Nursing Science Research Institute, College of Nursing, Daegu Catholic University, Daegu 42472, Republic of Korea

**Keywords:** infectious disease, nursing students, nursing program, virtual reality, respiratory infection

## Abstract

This study aimed to develop an immersive virtual reality (VR) program and conduct preliminary evaluation of its feasibility and learner perception for enhancing nursing students’ clinical practicum education. The VR program was designed using the ADDIE model (analysis, design, development, implementation, and evaluation) and implemented on the UNITY 3D platform. Expert evaluation was conducted through a VR application, and its effectiveness was further assessed among 25 fourth-year nursing students in terms of immersion, presence, and satisfaction. The expert evaluation yielded a mean score of 6.54 out of 7, indicating acceptable content validity. Among learners, evaluation demonstrated immersion at 42.28 ± 2.37 out of 50 (95% CI: 41.30–43.26), presence at 81.36 ± 7.32 out of 95 (95% CI: 78.34–84.38), and satisfaction at 13.48 ± 1.26 out of 15 (95% CI: 12.96–14.00). Overall, the developed VR program demonstrated acceptable expert validity and positive learner perceptions. These preliminary findings suggest feasibility as a supplementary practicum. However, the single-group design without control comparison and reliance on self-reported measures preclude conclusions about educational effectiveness.

## 1. Introduction

VR has garnered attention from medical educators in nursing education, aiming to reduce educational costs and risks while maintaining high standards of excellence [1,2,3]. As information technology rapidly advances, technologies like VR offer new pedagogical methods for nursing education [4]. VR provides nursing students an immersive and interactive environment by recreating real clinical scenarios, offering a powerful hands-on experience without direct patient contact. This approach not only saves valuable time for clinical nursing professionals but also alleviates challenges associated with traditional patient interactions in pedagogy [5], addressing the shortage of clinical education resources. Since the early 2000s, frequent outbreaks of emerging infectious diseases (IDs) have required nurses to effectively manage these patients to ensure patient safety. However, clinical practicums focus on observation rather than direct nursing care due to concerns about patient rights, which limits nursing students’ ability to meet the learning objectives of clinical practicum education [6]. To address these issues, nursing education has introduced simulation-based learning. VR simulation is more immersive than multimedia learning, resulting in better learning outcomes [7].

During the COVID-19 pandemic, nurses reported difficulties in caring for ventilated patients in isolated intensive care units (ICUs) and expressed the need for education to acquire knowledge and practical skills related to ventilators [8]. Education on ID knowledge, along with practice-oriented simulation training, alleviated the fear of caring for patients with infections and improved nursing competencies to prevent the transmission of IDs [9].

Although the COVID-19 pandemic has subsided, it could arise at any time. Therefore, it is crucial for nursing students to acquire knowledge and skills by caring for respiratory patients in preparation for future outbreaks of respiratory IDs. As millennials, nursing students have grown up in the digital age and require engaging, innovative teaching methods to foster their interest and enthusiasm for learning [10].

Previous studies targeting nursing students have predominantly focused on procedural learning such as blood transfusion, catheterization [11,12], donning protective equipment [13], and medication administration [14]. However, to adequately prepare for real-world practice, there is a critical need for scenarios that enable students to resolve complex events [13]. Beyond mastering procedures and techniques, there was a demonstrated need to develop practical nursing education programs for managing patients with respiratory infections in complex scenarios. Additionally, caring for patients with respiratory infections requires repeated practice to ensure both patient safety and healthcare provider safety due to infection risk. VR offers significant advantages over standardized or high-fidelity simulation by reducing educational costs, time constraints, and space limitations [15], making it an ideal platform for repeated practice. Therefore, it is essential to develop practical nursing education for comprehensive care of patients with respiratory infections in complex situations.

Building on the experiences of new nurses who dealt with COVID-19 [8] and a survey of nursing students’ educational needs regarding emerging respiratory IDs [16], we aimed to develop an immersive virtual reality (VR) educational program for nursing students and explore the process of developing it.

## 2. Methods

This research aimed to develop an immersive virtual reality (VR) program for nursing students and explore the process of developing it. We developed the VR program following the five steps of the analysis, design, development, implementation, and evaluation (ADDIE) model.

### 2.1. Analysis

Learner needs analysis utilized results from a survey conducted using a questionnaire developed through a literature review and consultation with six experts to identify educational needs for a program focused on emerging infectious diseases [16].

### 2.2. Design

The learning objectives were established based on the needs assessment (Table 1). We developed the scenarios following the guidelines of the International Nursing Association for Clinical Simulation and Learning (INACSL) [17], which recommend that simulation experiences should be designed in consultation with content experts as well as simulationists who are knowledgeable and competent in best practices in simulation education pedagogy and practice [17]. To meet the learning objectives, we developed three domains: (a) infection/contamination, (b) patient safety, and (c) patient care competency. The scenarios were designed to achieve the learning objectives as outlined in the scenario flowchart. We consulted two educational experts and two clinical experts caring for COVID-19 patients to verify the content’s accuracy.

**Figure 1:**
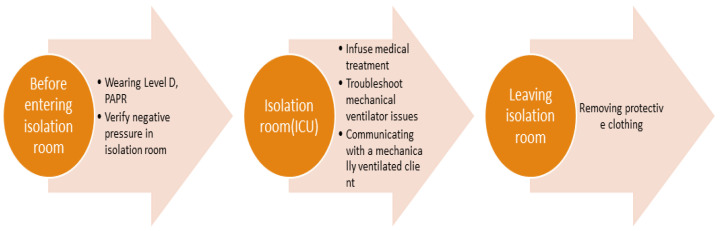
Scenario flowchart.

Scenarios followed the flowchart (Figure 1) with multimedia learning principles applied [18]: (1) Spatial contiguity principles: critical information (vital signs, PPE procedures) positioned adjacent to 3D objects. (2) Modality principles: voice narration synchronized with visual demonstrations rather than on-screen text. (3) Segmenting principles: scenarios divided into discrete phases (PPE donning → ICU assessment → PPE doffing). (4) Signaling principles: visual cues (glowing vital signs, highlighting next item in order) direct attention to key learning objectives.

The VR storyboard (Figure 1) sequences three environments: the protective equipment changing room → isolation ICU → protective equipment changing room. Learners progress as nurses from PPE donning, through patient assessment/care in ICU, to safe doffing, ensuring coherence by eliminating extraneous visual elements.

### 2.3. Development

The VR training platform was engineered using Unity 3D with biomedical engineering optimizations for clinical simulation. We utilized Oculus Quest 2 with dual handheld controllers, structuring content into practice and evaluation phases.

Key engineering contributions include the following: Human–computer interaction engineering: custom hand tracking interactions optimizing collision physics and input latency (<5 ms) for clinical psychomotor skill training [19]. Physiological simulation engineering: real-time virtual patient vital sign modeling integrated with learner interactions [20]. Cognitive engineering: multimedia learning principles (3D spatial contiguity, modality principle) embedded in spatial layout [21].

These engineering specifications enable immersive clinical practice while minimizing cybersickness through optimized HMD compatibility and lightweight controller interactions. The first-time user training requires 15–20 min, structured into practice and assessment phases.

### 2.4. Implementation

The developed VR program was implemented by nursing students.

### 2.5. Evaluation

The developed VR program was evaluated by the learner (nursing student) and the expert.

#### 2.5.1. Expert Evaluation

For the expert evaluation, we used the heuristic evaluation tool for VR applications (VRAs) developed by Sutcliffe and Gault [22]. It is recommended that the number of heuristic evaluators be between 3 and 5 [23]. The program was evaluated by two virtual reality program development experts and two nursing education experts with experience in the use of virtual reality programs. The VRA tool consists of 12 questions, each evaluated on a scale from 0 (no usability problem) to 4 (usability problem that must be fixed). If two or more experts assess a heuristic problem and rate its severity as 4 or higher, it is selected as an issue to be addressed in the improvements. The total score is then converted into a grade. We used the total score to identify which items had usability issues that needed fixing. The tool is scored on a scale of 1 to 7, where 1 denotes poor quality and 7 indicates excellent quality [22].

#### 2.5.2. Learner Evaluation

VR provides a high-presence and immersive environment that enhances learners’ psychomotor engagement, thereby facilitating more efficient and reliable cognitive learning processes and improving educational outcomes [24]. To evaluate the usability of the developed content, we assessed immersion, presence, and satisfaction. After using the VR educational content, learners completed a post-survey.

To assess learner convenience, this study received approval from the Institutional Review Board (IRB No. 23-01-0205) and collected data on 10 June 2023. The number of subjects for program evaluation was set at 25 based on a previous study [20], in which 20 nursing students evaluated a practical education application. Participants were fourth-year nursing students enrolled in a nursing college who had completed respiratory disease coursework and finished the first-semester curriculum. Participants were recruited through online social networking sites (SNSs) by explaining the study’s purpose and methods. There were 25 fourth-year nursing students who voluntarily consented to the training and survey on the day of the training.

To ensure participants’ ethical protection, a research assistant explained the study’s purpose, procedures, anonymity, and right to withdraw participation before conducting the survey. Participants were informed that the collected data would be coded and anonymized to maintain confidentiality, and then stored for three years before being destroyed.

Data were analyzed using SPSS version 21.0 to calculate frequencies, percentages, means, and standard deviations.

#### 2.5.3. Immersion

To assess immersion, we used the Flow Short Scale developed by Engeser and Rheinberg [25], which was translated into Korean and validated by Yoo and Kim [26]. The tool uses a 5-point Likert scale (1 = strongly disagree, 2 = disagree, 3 = neutral, 4 = agree, and 5 = strongly agree). The tool includes 10 questions, 6 related to performance proficiency and 4 related to immersion in a given activity, with higher scores indicating deeper immersion. The Cronbach’s α was 0.84 in Yoo and Kim [26] and 0.83 in this study.

#### 2.5.4. Presence

We assessed presence using a tool developed by Chung and Yang for 3D video evaluation [27]. The subdomains of this tool are perceived characteristics, impression, and presence. In this study, we used 19 items from the presence domain, with modifications to suit the study. These 19 items included 7 for spatial involvement, 4 for temporal involvement, 5 for immersive dynamism, and 3 for immersive presence, using a 5-point Likert scale (ranging from 1 = strongly disagree to 5 = strongly agree), where higher scores indicate a greater sense of presence. At the time of development, the tool had a Cronbach’s α ranging from 0.81 to 0.92 [27], and in this study, it was 0.85.

#### 2.5.5. Education Satisfaction

We assessed education satisfaction using an instrument developed by Cho [28] and adapted by Yu [29] for nursing students. The instrument consists of 3 questions rated on a 5-point Likert scale (1 = strongly disagree and 5 = strongly agree), where higher scores indicate greater education satisfaction. The reliability of the instrument at the time of development was indicated by a Cronbach’s α of 0.81, and in this study, it was 0.66.

## 3. Results

**Figure 2:**
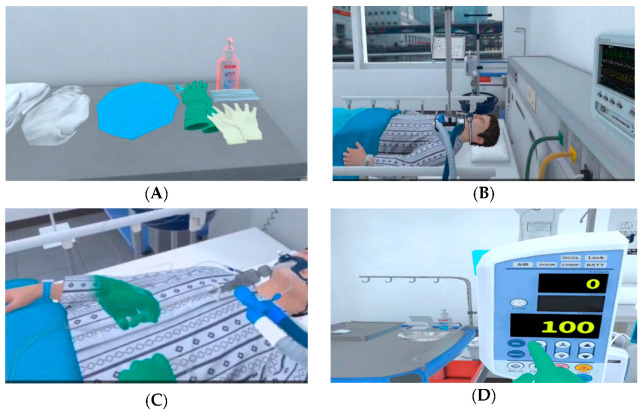
Contents of VR program. (**A**) Choose right protective equipment; (**B**) isolated ICU; (**C**) closed suction; (**D**) setting up infusion pump.

Learning objectives (1–10), established through learner needs assessment to enhance infection/contamination, patient safety, and patient care competencies, were implemented within scenarios as shown in Table 1. In the scenario, the patient is infected with COVID-19 and is in an isolated ICU. The nurse, wearing protective equipment, administers antiviral medications, troubleshoots ventilator issues, and safely removes the protective equipment. Achievement of learning objectives is confirmed through feedback provided upon scenario completion, with opportunities for learners to repeat and practice unachieved components. Content designed to enhance learner presence and immersion is presented in Figure 2.

### 3.1. Developed VR Program

The VR program enhanced immersion by providing users with various interactive opportunities through the use of a 3D VR headset with two handheld controllers (Oculus Quest 2). Visual feedback was provided using arrows and orange inversions (Figure 2A), while auditory feedback included real-world sounds, such as voice prompts for the next screen, sounds from real devices, and the sound of an automatic door opening, to create a realistic intensive care unit environment (Figure 2B). To enable tactile perception, the VR controller was used as a haptic device, allowing users to interact with displayed objects by touching them, such as closed suction (Figure 2C). When pressing the IV pump button, users could feel tactile stimuli, simulating the sensation of pressing a real button (Figure 2D), thereby maximizing tactile feedback. To extend the learner’s knowledge, they could calculate a dose or choose an answer to a patient’s question.

Learners could choose between a practice mode and an assessment mode. After sufficient practice, they could progress to the assessment mode to check their learning. At the end of the VR program, users could review their errors (e.g., the order of putting on and taking off protective equipment, incorrect quiz answers) on the final screen, maximizing the learning effect.

### 3.2. Implementation

Participants engaged with VR content for an average of 15–20 min. To ensure a safe learning environment, equipment was arranged to prevent overlapping pathways between students, and all obstacles that could lead to tripping or collisions were removed. For safety during VR use, students worked in pairs so that immediate assistance could be provided if anyone felt dizzy or needed help.

The sequence began with equipment orientation and mechanical usage training, followed by scenario content briefing before starting the VR experience. After the VR session, students took a 20 min break, completed questionnaires, and participated in structured debriefing.

### 3.3. Evaluation

#### 3.3.1. Learner Evaluation

After running the VR program, participants evaluated it. As shown in Table 2, participants scored 42.28 ± 2.37 out of 50 for immersion, 81.36 ± 7.32 for presence, and 13.48 ± 1.26 out of 15 for satisfaction.

#### 3.3.2. Expert Evaluation

The average score was 6.5, with each question ranging from 6 to 7 points. All four experts rated the content as usable, with suggestions to clearly highlight arrows pointing to objects and to slow down the speed of the user’s spatial movement, as fast movement could cause dizziness. The item “Close coordination of action and representation” received the highest ratings, while “Consistent departures” and “Clear turn taking” received the lowest ratings; however, the extent of required revision was deemed insufficient (Table 3).

## 4. Discussion

To improve nursing students’ ability to respond to patients with respiratory infections, we developed an immersive VR-based nursing program focused on patients with respiratory infections. It replicated the real environment of an isolation intensive care unit to enhance fidelity and was configured based on the needs survey to ensure that the complexity level was appropriate for fourth-year students. Additionally, appropriate cues were inserted during the program [18], which allowed learners to review related materials and integrate knowledge and experience through debriefing. The program was developed during the COVID-19 pandemic, a time when the need for prepared healthcare workers was urgent, driven by learners’ expectations and the demand for diverse educational experiences in a rapidly changing educational environment. We built a 3D model of an environment similar to an isolation room for patients with respiratory IDs, where learners could perform nursing care in each of the four areas using an Oculus Quest 2 head-mounted display and controller while taking quizzes to review relevant sections. While most existing immersive VR programs have been designed for procedural skill practice [11,12,13,14,30,31], this program differs by not only incorporating procedural learning but also preparing learners for entering an isolation ICU and providing care for critically ill, isolated patients. This expands the content beyond traditional infection control education for isolated patients [32].

The immersion level of the participants in this study was 42.28 out of 50 points. Immersion refers to the state of experiencing optimal effects by becoming deeply engaged in an activity. Learners experience immersion when the difficulty level of a task aligns with their sense of challenge [33]. High immersion likely resulted from the program being developed based on the learners’ educational needs [16], and their preparation for the challenge of caring for COVID-19 patients with respiratory infections in the ICU was enhanced through lectures, videos, and question-and-answer sessions during the pre-briefing period. This preparation helped balance task difficulty with challenge, facilitating participation in the program. Additionally, the headset and haptics stimulated the visual and tactile senses, enhancing immersion and providing feedback based on the learner’s interaction with objects [34]. This likely contributed to the greater immersion experienced by participants. Research supports this, suggesting that headset VR programs are more immersive because they use the body’s movements to create a more realistic experience [35].

However, learners participating in simulations may lose their sense of immersion when they feel they are being observed [36] or when they perceive the situation as unreal [37]. Furthermore, the high immersion rate in VR simulations may be due to learners’ autonomy. They can participate without being watched, can freely use the program at any time, and are motivated to learn through the engaging nature of the medium [12]. A previous study [31] also reported that realistic settings, where learners are completely disconnected from the real environment, enhance immersion. Furthermore, the ability to receive feedback on their performance likely motivated learning and increased immersion.

Novac [38] explained that presence is related to selective perception and attentional factors during the recognition phase and is associated with exploratory attention, which influences learning performance. In this study, the score for presence was 81 out of 95. Rhu [39] reported a mean score of 4.39 in a program involving care for neonatal ICU (NICU) patients, which is similar to the present study’s score of 4.26. This score is higher than the mean score of 130 out of 190 for the simple skill of transfusion in a prior study, which evaluated presence using a different tool. Both the previous study [12] and the present one show that presence is enhanced when spatial and temporal involvement—a sense of dynamism that encourages users to move their bodies—is combined with visual, auditory, and tactile elements that mimic the real world. To increase the realism of the program, we also used a 3D model of a real hospital setting, photographed medical devices and supplies, and augmented the presence of VR by allowing learners to move around the space and observe the objects [18]. Tactile presence was provided by using vibration when the learner operated an IV pump. Additionally, we employed realistic images and sounds associated with real items, such as mixing real TPN solutions, injecting an IV bolus through a real 3-way, and alarms from monitors or devices.

Education satisfaction with this program was 13 out of 15. As education satisfaction in VR programs is influenced by instructional design and immersion, it is essential to design instruction that promotes learners’ focus and engagement, as well as to select a medium that facilitates immersion [40]. A previous study also showed that learners who trained using VR were more satisfied with the training [41], supporting our findings. However, immersive VR education has side effects, such as difficulty operating the device and cybersickness [12,31,39], and some participants in our study reported issues with device operation and motion sickness. To minimize these challenges, it is essential to allow sufficient practice with the HMD and controllers before engaging with the VR program to prevent difficulties arising from inexperience.

The VR program in our study meets the needs of digital-native students and offers advantages such as reduced space requirements and ease of use compared to conventional simulation education. However, side effects may hinder its broader use in educational settings. Therefore, the program should be improved through a convergent approach between technology and education.

This study developed an immersive VR training program based on the nursing of patients with respiratory infections and evaluated its educational utility. This program goes beyond conventional, skill-focused VR training to include a recreation of the isolation intensive care unit environment and clinical decision-making elements, allowing participants to experience complex clinical situations. Results showed that learners’ engagement (42.28/50), sense of presence (81.36/95), and educational satisfaction (13.48/15) all achieved mid- to high-level scores. These results suggest that VR-based training can substantially contribute to developing future nursing professionals equipped to respond to infectious diseases.

First, the high level of engagement is closely related to the program’s design based on learner needs assessment. Learners experienced a balance of challenge and task difficulty as they pre-trained on complex tasks required for COVID-19 intensive care, such as donning and doffing personal protective equipment, responding to ventilator alarms, and administering antiviral medications. This finding is consistent with previous research demonstrating that the “challenge–skill balance” proposed by Flow theory maximizes engagement. Furthermore, the provision of multisensory feedback (visual, auditory, and tactile) enhanced the sense of realism and expanded the learner’s interactive experience, supporting previous research demonstrating the high immersive effects of headset-based VR.

Second, the high level of presence appears to be the result of a complex interplay of elements that enhance fidelity, including a 3D space that precisely models the actual clinical environment, auditory stimulation based on actual medical device sounds and alarms, and tactile stimulation utilizing vibration feedback. These multisensory interactive elements induce a sense of “presence” in the learner’s environment, consistent with reports that spatial and temporal engagement is particularly associated with improved learning outcomes. This relatively higher level of presence than that reported in existing simple technical VR programs underscores the importance of VR training based on complex clinical scenarios.

Third, the high level of training satisfaction is likely due to the program structure (practice mode–assessment mode) that enabled learner participation, immediate feedback on errors, and the practical clinical context of isolation ward nursing, which enhanced learning motivation. Previous studies have reported that VR training can increase learner interest and satisfaction and contribute to improved clinical performance, supporting the results of this study.

However, this study also identified side effects and operational limitations of immersive VR training. Some learners reported difficulty operating the Oculus Quest 2 or experiencing dizziness (cybersickness) due to inexperience with the device, a problem repeatedly raised in previous studies. In particular, movement speed, visual distance, and user adaptation are known to be factors inducing motion sickness, necessitating adjustments to the program’s movement speed and enhanced user guidance. Furthermore, because this program focused on a specific disease (COVID-19) based on a physical pandemic, its potential for expansion to various respiratory infection situations should be considered.

This study also has several limitations in terms of experimental design. First, the single-group posttest design makes it difficult to clearly verify the effectiveness of the VR program. Second, the limited number of learners (25) and the study being conducted at a single university limit the generalizability of the results. Third, because immersion, presence, and satisfaction were based on subjective assessment tools, we were unable to directly verify whether they actually improved clinical performance competency. Fourth, short exposure time precluded assessment of long-term effects.

Nevertheless, this study is significant in that it demonstrates the feasibility of developing a high-fidelity VR nursing education program that embodies high-quality clinical situations and integrates multisensory interaction based on a pre-assessment of learner needs in the high-risk and high-difficulty area of respiratory infection patient care. This demonstrates the potential for developing programs that transcend the limitations of existing skill-focused VR training and encompass complex competencies such as clinical thinking, communication, and patient safety management.

Future research should utilize a randomized controlled trial (RCT) design to verify the actual effectiveness of VR training in improving knowledge, skills, and clinical judgment compared to traditional lecture- or practice-based training. Furthermore, systematic analysis should be conducted to examine the impact of VR learning experiences on long-term knowledge retention, whether the benefits accumulate with repeated learning, and how to strengthen the debriefing structure. Finally, expanding the scenarios to include various infectious disease situations (e.g., RSV, SARS-like viruses, etc.) and incorporating AI-based adaptive feedback could create a more personalized learning environment.

## 5. Conclusions

This study aimed to develop an immersive VR program for nursing students, explore the process of developing it, and evaluate its usefulness in clinical practicum education. Pilot evaluation demonstrated moderate-to-high immersion, presence, and learner satisfaction among fourth-year nursing students. The program extends beyond conventional procedural VR training by integrating complex clinical decision-making and multisensory interaction. While promising for infection control education, future randomized controlled trials are essential to establish comparative effectiveness, long-term knowledge retention, and clinical performance outcomes relative to traditional simulation methods.

## Figures and Tables

**Table 1:** Study scenarios.

Domain	Numbers	Learning Objectives	Organization of Scenarios
Infection/contamination	1	Able to select Level D protective equipment and put it on in order.	The first scene shows putting on protective equipment. The last scene shows undressing.
	2	Able to operate a powered air-purifying respirator (PAPR) in the correct order.	
	3	Able to remove Level D protective equipment in the correct order.	
	4	Able to explain the basic design of a negative pressure room.	Entering the room after putting on equipment and checking the negative pressure in the isolation room.
Patient safety	5	Able to administer antiviral medication for patients with COVID-19.	Preparations of remdesivir, dexamethasone, and total parenteral nutrition (TPN) as prescribed and administered using an infusion pump.
	6	Able to operate an intravenous infusion pump to administer intravenous medications to patients with COVID-19.	
	7	Able to explain the ventilator settings.	Verifying the device settings for patients using ventilators and intervening during closed suction and e-tube cuff pressure based on alarm events.
	8	Able to troubleshoot alarms when applying a ventilator.	
Patient care competency	9	Able to communicate with a patient equipped with a ventilator.	A patient on a ventilator asks about release from isolation using a pen and note.
	10	Able to explain the release from isolation of a COVID-19 patient.	

**Table 2:** Variables after experience with VR program.

			(N = 25)
Variables	M ± SD	Min–Max	95%CI
Immersion	42.28 ± 2.37	34–50	41.30–43.26
Presence	81.36 ± 7.32	69–95	78.34–84.38
Satisfaction	13.48 ± 1.26	11–15	12.96–14.00

**Table 3:** Expert evaluation.

			(N = 4)
Variables	M ± SD	Min–Max	Range
Natural engagement	6.75 ± 0.50	6–7	1–7
Compatibility with the user’s task and domain	6.50 ± 0.57	6–7	1–7
Natural expression of action	6.50 ± 0.57	6–7	1–7
Close coordination of action and representation	7.00 ± 0.00	6–7	1–7
Realistic feedback	6.75 ± 0.50	6–7	1–7
Faithful viewpoints	6.50 ± 1.00	6–7	1–7
Navigation and orientation support	6.50 ± 0.57	6–7	1–7
Clear entry and exit points	6.50 ± 0.57	6–7	1–7
Consistent departures	6.25 ± 0.50	6–7	1–7
Support for learning	6.50 ± 0.57	6–7	1–7
Clear turn taking	6.25 ± 0.50	6–7	1–7
Sense of presence	6.50 ± 0.57	6–7	1–7

## Data Availability

Data are contained within the article.
